# A Comparison Between Two Different Directions of Landmark‐Guided Femoral Vein Puncture: A Prospective Randomized Controlled Trial

**DOI:** 10.1155/anrp/9638063

**Published:** 2026-04-16

**Authors:** Feng Liu, Xin Wang, Zhaoming Guan, Wang Shen, Longqiu Yang, Bing Tang

**Affiliations:** ^1^ Department of Anesthesiology, Shanghai East Hospital, Tongji University School of Medicine, Shanghai, 200120, China, tongji.edu.cn; ^2^ Department of Anesthesiology, Shengjing Hospital of China Medical University, Shenyang, 110004, Liaoning, China, cmu.edu.cn

**Keywords:** anatomical variation of femoral vasculature, femoral vein puncture, needle direction, vascular complications

## Abstract

**Background:**

Anatomic variations in the spatial relationship between the femoral vein and artery are recognized as predisposing factors for iatrogenic vascular injury during percutaneous procedures. There have been few clinical studies comparing different directions of femoral vein puncture. Here, we conducted a prospective randomized controlled trial comparing two directions of femoral vein puncture.

**Methods:**

A cohort of patients undergoing percutaneous femoral venous catheterization was randomized into two groups of comparable size, with punctures executed via either the lateral or orthogonal approach. The primary outcome was the first‐attempt success rate. Secondary outcomes included overall success rate, number of puncture attempts, puncture time, and complications.

**Results:**

Among 108 patients, the lateral approach group demonstrated a higher first‐attempt success rate (76% vs. 52%; risk difference: 24%, 95% CI: 6% to 40%; *p* = 0.009) and overall success rate (91% vs. 61%; risk difference: 30%, 95% CI: 14% to 45%; *p* < 0.001), fewer puncture attempts (*p* = 0.004), and a shorter puncture time (median difference: −6.0 s, 95% CI: −13.0 to 0.0; *p* = 0.042). The advantages of the lateral approach were more significant when there was a lumen overlap between the femoral vein and artery. There were similar complication rates between approaches.

**Conclusions:**

When relying on anatomical landmarks for femoral vein puncture, the lateral approach is conducive to improving the success rate and reducing operating time without raising the risk of local complications. This is particularly beneficial in cases where the femoral artery and vein partially or fully overlap.

**Trial Registration:** Chinese Registry of Clinical Trials: ChiCTR2500096775

## 1. Introduction

In addition to transporting blood, the femoral vein (FV) acts as a conduit for blood products, resuscitative fluids, total parenteral nutrition administration, drugs, vascular intervention procedures, and mechanical extracorporeal therapies [1–3]. Femoral venous catheterization demonstrates elevated risks of catheter‐related bloodstream infections secondary to anatomical proximity to perineal flora, alongside an increased thrombotic incidence attributed to catheter migration–induced vascular endothelial damage [4, 5]. While the internal jugular vein (IJV) and subclavian vein (SCV) are commonly utilized as alternative routes for vascular access, there are specific benefits to utilizing the FV in certain clinical scenarios. For example, its distance from the atrium increases safety, and its large lumen decreases the risk of collapse and allows for the insertion of longer catheters that are less likely to dislodge [6]. These advantages are significant in anesthesia, critical care, pediatrics, and interventional medicine. With the advancement of cardiac catheterization procedures, the FV may become the preferred puncture site [7].

However, femoral arteriovenous topographic variability demonstrates an increased predisposition to vascular access–related iatrogenic injuries. A previous study conducted by Baum et al. reported that the spatial relationship between the femoral artery (FA) and FV at the inguinal ligament level presents substantially greater anatomical complexity than illustrated in canonical anatomical references [8]. While previous studies have reported the prevalence and classification of femorovascular anatomical variations within the inguinal region, information about different directions of FV puncture is scarce. Therefore, we designed and executed a prospective randomized controlled trial comparing two different directions of FV puncture in the landmark‐guided method. We hypothesized that compared with the orthogonal approach, the lateral approach would enhance success probability, minimize puncture attempts, and conserve procedural time.

## 2. Methods

### 2.1. Research Ethics

This was a prospective, randomized, superiority trial. Ethical approval was granted by the Ethics Committee of Shanghai East Hospital on December 12, 2024 (Approval No. 2024YS‐280). The trial was prospectively registered with the Chinese Clinical Trial Registry on February 6, 2025. For elective patients, written informed consent was obtained prior to randomization. For emergency or critically ill patients who were unable to provide immediate consent, written informed consent was obtained from their legally authorized representatives before study procedures. All enrolled participants or their legal representatives provided written informed consent. The detailed study protocol is available as Supporting Information 1.

### 2.2. Patient Inclusion and Exclusion

**FIGURE 1:**
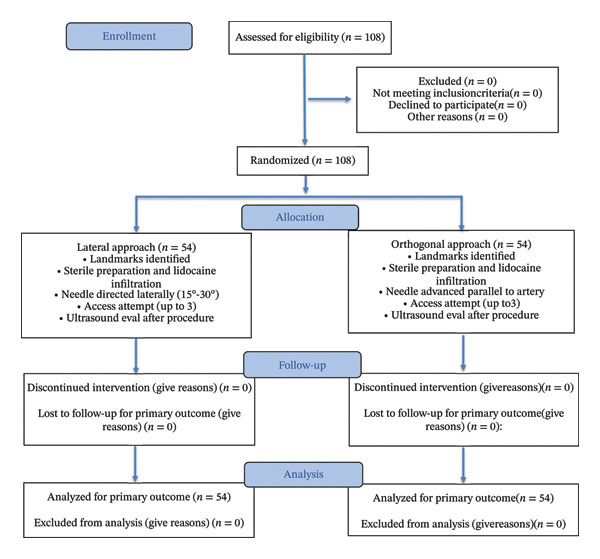
CONSORT diagram of the study.

The flow of participants through the trial is shown in Figure 1. A total of 108 adult patients aged ≥ 18 years (ASA physical status: I‐IV) who underwent FV puncture and catheterization in our hospital from February 22, 2025, to July 30, 2025, were included in this study. Exclusion criteria encompassed: (1) thrombotic or inflammatory involvement of target vasculature; (2) localized infections at puncture sites (e.g., tinea cruris); (3) prior femoral/pelvic vascular surgery; (4) preexisting inguinal hernia; and (5) peripheral artery disease (PAD). All procedures in this study utilized standard two‐lumen central venous catheters (Arrow International LLC). The clinical indications for central venous access included perioperative monitoring, fluid resuscitation, and vasoactive drug administration. And all procedures were performed in the operating rooms of the Department of Anesthesiology, Shanghai East Hospital. The CONSORT 2010 checklist is provided as Supporting Information 2.

### 2.3. Randomization and Group Allocation

**FIGURE 2:**
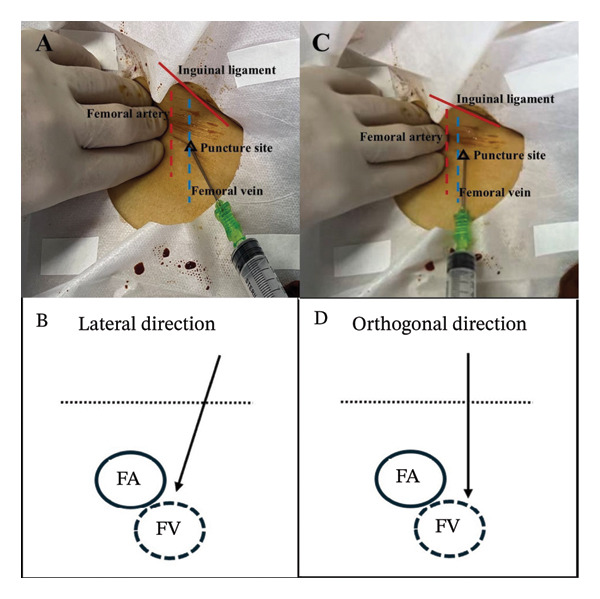
Types of puncture needle directions. Note: Solid red line, inguinal ligament; dashed red line, femoral artery; dashed blue line, femoral vein; black triangle, puncture site. The figure uses simplified line drawings to indicate the surface projections of anatomical structures, intended solely to illustrate differences in puncture direction. In clinical practice, the femoral artery and vein are not simply parallel; they lie at different depths, form an angle, and exhibit varying degrees of overlap. Abbreviations: FA: femoral artery; FV: femoral vein.

The experimental group adopted the lateral approach method, with the puncture site located medial to the FA, but the needle was directed laterally at a medial‐to‐lateral angle of 15°–30° (Figures 2(a) and 2(b)). The control group used the orthogonal approach at the same needle entry point as the experimental group, but advanced the needle parallel to the FA (Figures 2(c) and 2(d)). Procedures were performed by one of three operators with significant femoral access experience (range: 3–8 years; median: 5 years; all > 50 prior successful punctures). Patients were randomly assigned in a 1:1 ratio to either the experimental group or control group using a computer‐generated sequence with block randomization (block size = 6). Subsequently, within each group, patients were further randomized to one of three operators (1:1:1 allocation) through a nested block design. An independent statistician generated all sequences, and allocation concealment was maintained via a central web‐based system. Operators were notified of group assignment only after patient enrollment. This allocation concealment procedure was implemented through a central web‐based system that allowed for immediate randomization upon patient arrival, making it equally feasible for both elective and emergency procedures. A total of 108 consecutive patients who met the inclusion criteria were enrolled during the study period. All patients completed the study procedures and were included in the final analysis.

### 2.4. Procedure

During the access procedure, patients were placed in a supine position with slight external rotation of the hips (the “frog‐legged” position). Anatomic reference points (iliac crest, pubic tubercle, and inguinal ligament) were delineated via palpation. The FA pulsation (2–3 cm caudal to the ligament midpoint) guided the puncture site (1–2 cm medial to the artery).

The standardized protocol included (1) aseptic preparation; (2) 2% lidocaine 5 mL infiltration; (3) needle insertion at 30°–45° cephalad according to randomization; (4) timing started at skin breach; (5) chronometer stopped upon successful access or after 3 failed attempts; and (6) ultrasound guidance initiated after 3 failures. Of note, ultrasound guidance was not used as the initial approach for any patient; it was reserved solely as a rescue technique after three unsuccessful landmark‐guided attempts. An “attempt” was defined as a discrete skin puncture event, continuing until successful access or termination due to patient risk.

All punctures were performed on the right FV. For patients under general anesthesia (*n* = 60), the procedure was performed intraoperatively. For awake patients (*n* = 48), the procedure was performed in the operating room under local anesthesia with standard monitoring. To minimize bias, all procedures followed a standardized operating procedure, with critical steps documented by an independent observer and video recordings reviewed retrospectively. The standard operating procedure is detailed in Supporting Information 3.

**FIGURE 3:**
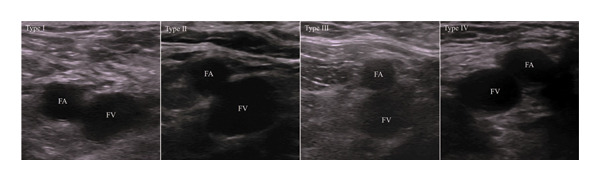
Topographical classifications of femoral arteriovenous spatial relationships. Note: Type I: parallel venoarterial orientation, no overlap. Type II: medial venous position, ≤ 50% luminal overlap. Type III: medial venous position, > 50% overlap. Type IV: lateral venous course. All ultrasound images presented in the figure were derived exclusively from study participants, with documented informed consent obtained. Abbreviations: FA: femoral artery; FV: femoral vein.

Guan et al. delineated femorovascular anatomy into four types based on spatial relationships (Figure 3) [9]. Vascular ultrasonographic evaluation of femoral vasculature anatomy was implemented after catheterization, focusing on spatial relationships within the puncture zones.

### 2.5. Outcome Measurement

The primary outcome of this study was the first‐attempt success rate of FV puncture. Secondary outcomes included the overall success rate, the number of puncture attempts, puncture time, and complications. Femoral vessel anatomical types were recorded as covariates for subgroup analysis.

### 2.6. Sample Size Calculation

PASS 2021 (NCSS, East Kaysville, UT, USA) was employed for sample size computation. The sample size calculation was based on the primary outcome. A small, nonblinded, unpublished pilot study with 20 participants indicated that the first‐attempt success rates for the lateral and orthogonal approaches were 90% and 60%, respectively. Based on the pilot data, we hypothesized that the lateral approach would be superior to the orthogonal approach in first‐attempt success rate. With a two‐sided error of 0.05 and a power of 0.9, the sample size for each group was calculated as 42. To account for 20% attrition, we increased the sample size to 54 per group. Details of the pilot study are presented in Supporting Information 4.

### 2.7. Statistical Analysis

Statistical analysis was conducted using SPSS v27.0 (SPSS Inc., Chicago, IL, USA). Normality and variance homogeneity were assessed using the Shapiro–Wilk test and Levene’s F‐test, respectively. Continuous variables showing a normal distribution were summarized as mean ± SD and compared across groups using independent Student’s *t*‐tests. Continuous variables with a nonnormal distribution were expressed as median (IQR) and compared using the Mann–Whitney *U* test. Categorical variables were reported as frequencies (%) and evaluated with Pearson’s *χ* ^2^ tests. As this was a superiority trial, the primary outcome (first‐attempt success rate) was analyzed using risk difference with its 95% confidence interval. Risk differences were calculated using the Newcombe method (Wald with continuity correction). The same approach was applied to secondary binary outcomes. Time‐to‐event outcomes were illustrated using Kaplan–Meier curves. For a post hoc sensitivity analysis, we employed a logistic regression model. Statistical significance was defined as *p* < 0.05.

## 3. Results

### 3.1. Patients

Throughout the study duration, 108 patients were successfully recruited and underwent right FV puncture and catheterization: 54 were allocated to the experimental group (lateral approach) and 54 to the control group (orthogonal approach). All patients completed the study procedures and were included in the final analysis. As shown in Table 1, no significant intergroup differences were observed in demographic parameters (age, gender, and BMI), ASA physical status classification, comorbidity profiles, or anesthesia type (general versus other).

**TABLE 1:** Patient characteristics.

	**Lateral approach (*n* = 54)**	**Orthogonal approach (*n* = 54)**	**p**

Age, yr	59.2 ± 13.2	57.7 ± 12.6	0.548
Gender (male/female)	39/15	31/23	0.107
BMI, kg m^−2^	21.0 ± 2.1	20.8 ± 2.3	0.518
ASA (I/II/III/IV)	0/4/35/15	0/3/35/16	0.916
Hypertension, *n* (%)	19 (35.2)	23 (42.6)	0.430
Hypotension/shock, *n* (%)	5 (9.3)	8 (14.8)	0.375
Diabetes, *n* (%)	20 (38.5)	17 (31.5)	0.451
Coronary artery disease, *n* (%)	11 (20.4)	7 (13.0)	0.302
Stroke, *n* (%)	1 (1.9)	3 (5.6)	0.308
Atrial fibrillation, *n* (%)	0 (0)	2 (3.7)	0.475
Coagulation disorders, *n* (%)	16 (29.6)	12 (22.2)	0.380
Preexisting coagulopathy, *n* (%)	2 (3.7)	1 (1.9)	0.558
Anticoagulant therapy, *n* (%)	14 (25.9)	11 (20.4)	0.494
Under general anesthesia, *n* (%)	35 (64.8)	25 (46.3)	0.053

*Note:* yr, year.

Abbreviations: ASA, American Society of Anesthesiologists; BMI, body mass index.

### 3.2. Success Rate

Technical success rates are summarized in Table 2. Primary outcome analysis revealed that the first‐attempt success rate in the lateral approach group was 76% (41/54), significantly higher than 52% (28/54) in the orthogonal approach group, with a statistically significant difference (risk difference: 24%, 95% CI: 6% to 40%; *p* = 0.009), indicating superiority of the lateral approach. Secondary outcome analyses showed that the overall success rates were 91% (49/54) in the lateral approach group and 61% (33/54) in the orthogonal approach group, showing a statistically significant difference (risk difference: 30%, 95% CI: 14% to 45%; *p* < 0.001). The lateral approach puncture group had fewer puncture attempts, particularly when the anatomical relationship between the FA and FV was classified as Type III.

**TABLE 2:** Results: success rate, puncture time, anatomical relationship, and complications.

	**Lateral approach (*n* = 54)**	**Orthogonal approach (*n* = 54)**	**p**

First‐attempt success rate	41 (76 [63 to 85])	28 (52 [39 to 65])	0.009
Overall success rate	49 (91 [80 to 96])	33 (61 [48 to 73])	< 0.001
Puncture time, median (IQR), s	49.5 (45.0, 60.0)	59.0 (46.0, 78.3)	0.042
Number of attempts (1/2/3)	41/4/9	28/3/23	0.004
1	41 (76 [63 to 85])	28 (52 [39 to 65])	0.009
2	4 (7 [3 to 18])	3 (6 [2 to 15])	0.696
3	9 (17 [9 to 29])	23 (43 [30 to 56])	0.003
Crossover to ultrasound‐guided	5 (9 [4 to 20])	21 (39 [27 to 52])	< 0.001
Anatomical relationship (I/II/III/IV)	4/32/16/2	5/31/18/0	0.523
Type I	4 (7 [3 to 18])	5 (9 [4 to 20])	0.728
First‐attempt success rate	4 (100.0)	5 (100.0)	—
Overall success rate	4 (100.0)	5 (100.0)	—
Puncture time, median (IQR), s	38.5 (35.0, 55.5)	44.0 (39.5, 46.5)	0.462
Number of attempts (1/2/3)	4/0/0	5/0/0	—
Type II	32 (59 [46 to 71])	31 (67 [44 to 70])	0.845
First‐attempt success rate	28 (88 [72 to 95])	23 (74 [57 to 86])	0.179
Overall success rate	31 (97 [84 to 99])	28 (90 [75 to 97])	0.583
Puncture time, median (IQR), s	49.0 (46.0, 57.0)	51.0 (45.0, 66.0)	0.831
Number of attempts (1/2/3)	28/2/2	23/3/5	0.375
Type III	16 (30 [19 to 43])	18 (33 [22 to 47])	0.679
First‐attempt success rate	9 (56 [33 to 77])	0 (0/18)	< 0.001
Overall success rate	14 (88 [64 to 97])	0 (0/18)	< 0.001
Puncture time, median (IQR), s	53.5 (42.3, 76.0)	78.0 (74.3, 84.3)	0.001
Number of attempts (1/2/3)	9/2/5	0/0/18	< 0.001
Type IV	2 (4 [1 to 13])	0 (0)	0.475
First‐attempt success rate	0 (0/2)	—	—
Overall success rate	0 (0/2)	—	—
Puncture time, s	69, 82	—	—
Number of attempts (1/2/3)	0/0/2	—	—
Complication			
Femoral artery puncture	5 (9 [4 to 20])	2 (4 [1 to 13])	0.434
Hemorrhage/hematoma	2 (4 [1 to 13])	1 (2 [0 to 10])	0.558

*Note:* Data are presented as *n* (% [95% CI]) for categorical variables. The em dash (—) indicates that the data were clinically insignificant or that analysis was precluded by methodological constraints. For Type IV anatomy (*n* = 2, both in the lateral approach group), puncture times are presented as individual values.

Abbreviations: CI, confidence interval; IQR, interquartile range.

Table 2 also shows the anatomical type subgroup analysis. Among patients with Type II anatomical configurations, the lateral approach group achieved a first‐attempt success rate of 88% (28/32) and an overall puncture success rate of 97% (31/32). In contrast, the orthogonal approach group had success rates of 74% (23/31) for first‐attempt and 90% (28/31) overall. However, the differences were not statistically significant (risk difference: 13%, 95% CI: −7% to 33%, *p* = 0.179; risk difference: 7%, 95% CI: −8% to 22%, *p* = 0.583). Among patients with Type III anatomical configurations, the lateral approach group demonstrated significantly higher overall puncture success rate (88% vs 0%; risk difference: 88%, 95% CI: 64% to 97%; *p* < 0.001) and superior first‐attempt success rate (56% vs 0%; risk difference: 56%, 95% CI: 33% to 77%; *p* < 0.001) compared to the orthogonal approach group.

### 3.3. Puncture Time

**FIGURE 4:**
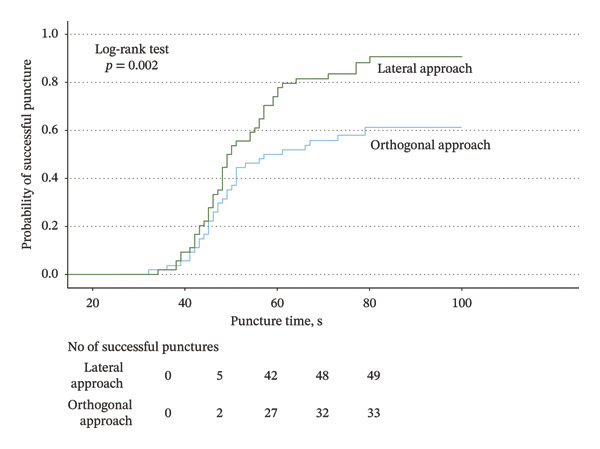
Kaplan–Meier curve showing time to successful femoral vein puncture. Note: Patients who were switched to ultrasound guidance after three failed attempts were censored at the time of their last attempt. The *x*‐axis starts at 20 s, as no puncture was achieved within 20 s.

The puncture time differed between the two groups. As shown in Table 2 and Figure 4, the lateral approach group had a statistically significantly shorter puncture time (median difference: −6.0 s, 95% CI: −13.0 to 0.0, *p* = 0.042). The difference between the two groups is primarily due to Type III anatomical relationships, which lead to an elevated success rate and reduced puncture attempts in the lateral approach, resulting in more efficient use of puncture time (median difference: −23.5 s, 95% CI: −34.0 to −8.0, *p* = 0.001).

### 3.4. Complications

Regarding vascular access complications, FA puncture occurred in five patients (9%) in the lateral approach group and two patients (4%) in the orthogonal approach group; however, this difference was not statistically significant (risk difference: 6%, 95% CI: −5% to 17%; *p* = 0.434). Hemorrhage or hematoma was observed in two patients (4%) in the lateral approach group and one patient (2%) in the orthogonal approach group, also without statistical significance (risk difference: 2%, 95% CI: −7% to 11%; *p* = 0.558). No other complications, such as pseudoaneurysm, were observed in either group.

### 3.5. Sensitivity Analysis

**FIGURE 5:**
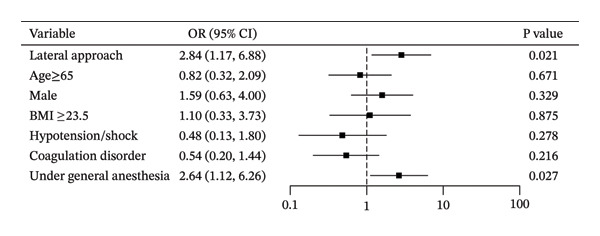
Post hoc sensitivity analysis using multivariable logistic regression. Note: the *x*‐axis was log10 transformed. Abbreviations: ASA: american society of anesthesiologists; BMI: body mass index; CI: confidence interval; OR: odds ratio.

Multivariable logistic regression analysis was conducted to evaluate predictors of FV puncture success, incorporating demographic parameters (age, BMI, and gender), key comorbidities (hypotension and coagulation disorder), anesthetic modality (general vs. other), and puncture direction (lateral vs. orthogonal). The advantage of the lateral approach on the puncture success rate remained after analysis (OR = 2.84, 95% CI: 1.17 to 6.88, *p* = 0.021) (Figure 5). Because the outcome was not rare (first‐attempt success rate in the orthogonal group was 52%), the odds ratio may overestimate the relative effect; therefore, the results of the logistic regression should be interpreted with caution. We further explored the outcomes stratified by anesthesia type within each puncture approach. The results are summarized in Supporting Information 5 (Supporting Table 1).

## 4. Discussion

This study is the first to have specifically analyzed the safety and efficacy of different needle directions in landmark‐guided puncture techniques of the FV. In emergencies such as cardiac arrest, central venous catheterization can be performed in the groin area without interrupting cardiopulmonary resuscitation [1, 10]. The FV may also be the preferred location for children undergoing cardiac surgery [11–13].

The superiority of the lateral approach is attributed to the anatomical variation of femoral vessels [14]. Guan et al.’s retrospective ultrasound analysis (*n* = 254) identified distinct anatomic distributions: upper inguinal level predominantly showed Type II (92.5%), followed by Type I (6.5%), with rare variants (Type III: 0.6% and Type IV: 0.4%); lower inguinal anatomy revealed Type II predominance (70.7%) with elevated Type III (23.4%) and IV (5.9%) [9]. Furthermore, Lee et al.’s retrospective abdominopelvic CT analysis (*n* = 584) identified a proximal‐to‐distal gradient in FA–FV overlap, with progressive overlap intensification toward the distal segments [15]. The survey of children’s inguinal regions conducted by Bhatia et al. yielded similar results [16]. We find that the lateral approach puncture is more appropriate for cases where the FA and FV overlap, in improving the success rate and reducing puncture time. When the overlap is substantial (Type III), the advantages of the lateral approach puncture become even more apparent.

Femoral puncture–related adverse events predominantly stem from accidental puncture of the FA [1]. Although the lateral approach did not significantly increase the incidence of complications compared to the orthogonal approach, the clinical implications of even rare complications warrant careful consideration in clinical practice. It should be noted that peripherally inserted central catheters (PICCs) demonstrate significantly lower mechanical complication rates and superior suitability for prolonged use, particularly in noncritical care settings [17, 18]. While PICCs were not included in our study population, we acknowledge their growing role in elective settings.

The crossover rate to ultrasound guidance was significantly higher in the orthogonal approach group (*n* = 21) than in the lateral approach group (*n* = 5) (*p* < 0.001). All crossover procedures resulted in successful venous access. Compared to landmark‐guided puncture techniques, ultrasound‐guided FV puncture significantly enhances the puncture success rate and lowers vascular injury risks [19, 20]. The Society of Hospital Medicine (SHM) advises physicians to use real‐time ultrasound guidance to establish FV access [21]. Although Type IV anatomical variants are rare, landmark‐guided techniques cannot achieve successful puncture in these cases; therefore, ultrasound‐guided puncture becomes mandatory.

The FV is the preferred puncture site for cardiac catheterization procedures [22, 23]. The lateral puncture approach involves the operator locating the position and depth of the FA through palpation. It requires greater operator skill. Moreover, there may be anatomical differences between the bilateral inguinal regions [24]. Factors such as underlying diseases and body surface area may influence femoral vein diameter (FVD) [25]. Adequate clinical expertise, understanding of procedural workflow, and manual dexterity are essential requirements for operators to ensure safe femoral venous catheterization. Therefore, we recommend that medical personnel primarily use ultrasound real‐time guided puncture. For operators familiar with FV puncture and catheterization, the lateral approach can be utilized to enhance the success rate and reduce puncture time when ultrasound equipment is unavailable. For inexperienced operators, if the FV cannot be located using the orthogonal approach during an emergency, the lateral approach may be attempted.

This study has several limitations. No significant intergroup differences in complication rates were found in the current sample, which may lack the power to reveal true associations, emphasizing the need for more extensive research. Additionally, despite utilizing independent assessors for outcome verification, operator‐controlled variables (e.g., needle advancement speed or pause frequency) could systematically differ between groups with the nonblinded nature of the procedure. Another limitation is the absence of stratification by vascular anatomical type during randomization. This was due to the intentional avoidance of preoperative ultrasound to prevent operator bias and the marked disparity in subtype prevalence (e.g., extreme rarity of Type IV anatomy). Future studies with larger cohorts or dedicated multicenter collaborations should consider prospective stratification to specifically evaluate outcomes across rare anatomical subtypes.

## 5. Conclusion

Our study indicates that when relying on anatomical landmarks for FV puncture and catheterization, the lateral approach puncture is conducive to improving the success rate, reducing puncture attempts, and saving procedural time, particularly in cases where there is a partial or complete overlap between the FA and FV.

## Author Contributions

Bing Tang and Longqiu Yang supervised the project. Feng Liu and Xin Wang contributed to manuscript drafting. Feng Liu, Xin Wang, Zhaoming Guan, and Wang Shen helped in acquiring and sorting the data. Feng Liu and Xin Wang helped in analyzing and interpreting statistics. Longqiu Yang, Feng Liu, and Xin Wang helped in preparing the figures. All authors were involved in drafting and reviewing the manuscript.

## Funding

This study was supported by the New Quality Clinical Specialty Program of High‐End Medical Disciplinary Construction in Shanghai Pudong New Area (2024‐PWXZ‐02) and Pudong New Area Health System Leading Talent Training Program (PWR12024‐07).

## Disclosure

All authors approved the final manuscript for submission.

## Ethics Statement

This clinical study was approved by the Institutional Review Board and Ethics Committee of Affiliated East Hospital of Tongji University (No: 2024YS‐280), Shanghai, China (Chairperson Prof Zengguang Xu). All patients signed written consent form to participate in this study. This study was conducted in accordance with the Helsinki Declaration‐2013.

## Consent

Please see the Ethics Statement.

## Conflicts of Interest

The authors declare no conflicts of interest.

## Supporting Information

Additional supporting information can be found online in the Supporting Information section.

## Supporting information


**Supporting Information 1** Clinical study protocol: The detailed study protocol outlining the trial design, methodology, inclusion/exclusion criteria, and outcome measures.

**Supporting Information 2** CONSORT 2010 flow diagram: A CONSORT 2010–compliant flow diagram is provided to systematically illustrate participant progression through the randomized controlled trial (RCT).

**Supporting Information 3** Standard operating procedure (SOP): The standardized procedure protocol used to ensure consistent execution and data collection across all trial participants.

**Supporting Information 4** Preliminary exploratory study: The pilot study that preceded and guided the design of the main trial.

**Supporting Information 5** Subgroup analysis of procedural outcomes stratified by puncture approach and anesthesia type.

## Data Availability

The data that support the findings of this study are available from the corresponding author, Bing Tang, upon reasonable request.
