# Supplementary material for: A Comparison Between Two Different Directions of Landmark‐Guided Femoral Vein Puncture: A Prospective Randomized Controlled Trial
Source: Anesthesiol Res Pract. 2026 Apr 16;2026:9638063. doi: 10.1155/anrp/9638063 (PMC13267157; doi:10.1155/anrp/9638063)
Supplement: Supplementary file 1 — Supporting Information 1 Clinical study protocol: The detailed study protocol outlining the trial design, methodology, inclusion/exclusion criteria, and outcome measures. [file ANRP-2026-9638063-s001.pdf]

Clinical Study Protocol

TITLE:
A Comparison of Success Rates and Complications between Different needle insertion approaches for Landmark-Guided Femoral Vein Cannulation
A Prospective Randomized Controlled Trial

PRINCIPAL INVESTIGATOR: Feng Liu, Xin Wang, Bing Tang, Longqiu Yang, Wang Shen, Zhaoming Guan, Xiuqin Yu, Dan Su, Tao Liang, Jixiong Sun.

PROTOCOL SYNOPSIS

Title: A Comparison of Success Rates and Complications between Different needle insertion approaches for Landmark-Guided Femoral Vein Cannulation

Study Type: A prospective, single-center, Randomized Controlled, Clinical Trial

Corresponding Author: Bing Tang, Department of Anesthesiology, Shanghai East Hospital, Tongji University School of Medicine, Shanghai, China. Longqiu Yang, Department of Anesthesiology, Shanghai East Hospital, Tongji University School of Medicine, Shanghai, China.

Study Center: Shanghai East Hospital, Tongji University School of Medicine

Ethics: Approved by the Ethics Committee of Shanghai East Hospital (Approval No. 2024YS-280).

Trial Registration: www.chictr.org.cn (ChiCTR2500096775)

CONTENTS

I. STUDY OBJECTIVES 3

II. BACKGROUND 3

A. Challenges of Femoral Vein Catheterization 3

B. Anatomical Overview of the Femoral Vein 3

C. Preliminary Studies 3

III. METHODS 4

A. Recruiting Methods....................4

B. Inclusion Criteria 4

C. Exclusion Criteria 4

D. Consent Procedure....................4

E. Randomization and Blinding....................5

F. Sample Size Calculation 5

G. Statistical Analysis 5

H. Intervention and Study Procedures....................5

I. Measurements and Endpoints 6

IV. DATE AND SAFETY MONITORING....................6

V. FUNDING....................6

VI. INFORMATION CONFIDENTIALITY 6

VII. LITERATURE CITED....................7

I. STUDY OBJECTIVES

Central venous catheterization is a critical clinical procedure for diagnostics and treatments such as fluid resuscitation, blood transfusion, chemotherapy, and central venous pressure monitoring, and is widely used in critically ill patients [1, 2]. Currently, there is a lack of systematic research and statistical analysis regarding the efficacy and safety of different needle insertion approaches for landmark-guided femoral vein puncture. This study aims to compare the success rates and safety of different needle insertion directions during landmark-guided femoral vein puncture, aiming to provide references and inspire new insights for healthcare practitioners in clinical practice.

II. BACKGROUND

A. Challenges of Femoral Vein Catheterization

Among the commonly used central venous access routes, the femoral vein is a vital option, particularly recommended when superior routes are obstructed. It offers advantages like a large lumen and stability in hypovolemic patients [3]. However, its main challenge lies in the high anatomical overlap between the femoral artery and vein within the femoral triangle, which can exceed 70% just 2 cm below the inguinal ligament [4]. This overlap significantly increases the risk of complications—such as arterial puncture, hematoma, infection, and thrombosis—during the commonly used "blind palpation" or landmark-guided technique [5]. Despite the clear superiority of ultrasound guidance endorsed by major medical societies, its availability is often limited in real-world clinical settings, especially in emergencies, primary care hospitals, and under-resourced regions. Therefore, mastering and optimizing the traditional landmark technique remains a necessary skill for clinicians, underscoring the pressing need to improve its safety and efficacy.

B. Anatomical Overview of the Femoral Vein

The conventional landmark technique relies on palpating the femoral artery as a reference, with the standard puncture point being 2-3 cm inferior to the inguinal ligament and 0.5-1.0 cm medial to the arterial pulse, advancing the needle parallel to the artery (a "medial and parallel" approach) [6, 7]. Given the high prevalence of the femoral vein lying directly beneath the artery (classified as Types II and III by Guan et al.), this traditional anterior approach may frequently miss the vein or risk traversing the artery [8]. To address this anatomical challenge, an alternative "lateral-medial" needle direction has been proposed [9]. In this approach, the needle is inserted from the same medial starting point but is angled towards the femoral artery. The rationale is that this lateralward trajectory may better target the vein when it is situated posteriorly (in a deep position) relative to the artery, potentially improving the first-pass success rate and reducing the number of attempts and associated complications.

C. Preliminary Studies

Currently, there is a lack of robust, comparative studies systematically evaluating the efficacy and safety of different needle insertion directions for landmark-guided femoral vein puncture. To fill this evidence gap, our study is designed as a prospective randomized controlled trial. We will compare the traditional "medial and

parallel" approach with the innovative "lateral-medial" approach in patients requiring landmark-guided femoral vein cannulation. Key outcomes measured will include the first-attempt success rate, overall success rate, number of puncture attempts, procedural time, and the incidence of immediate complications. By providing high-level evidence on the optimal needle direction, this research aims to establish a simple, no-cost modification to a fundamental clinical skill. The findings are expected to offer valuable guidance for anesthesiologists, intensivists, and emergency physicians, ultimately enhancing patient safety in diverse clinical environments where ultrasound is not immediately available.

III. METHODS

This is a prospective randomized, controlled trial that has been registered in the Chinese Clinical Trial Registry (ChiCTR2500096775) and has been approved by the Ethics Committee of Shanghai East Hospital (Approval No. 2024YS-280). This report adheres to the CONSORT guidelines. The study was conducted according to the guidelines of the Declaration of Helsinki with Good Clinical Practice. All participants provided written informed consent before enrolment.

A. Recruiting Methods

Potential participants to the study were identified from the elective surgery list. Patients aged ⩾18 years, ASA I to IV, and undergoing femoral vein puncture and catheterization in our hospital were eligible to participate. The study will involve the use of protected health information. The study site will gain permission from each subject to use their protected health information by written authorization. All subjects will be identified by the anesthesiologist.

B. Inclusion Criteria

Patients aged ⩾18 years, ASA I to IV, and undergoing femoral vein puncture and catheterization in our hospital were eligible to participate.

C. Exclusion Criteria

Exclusion criteria encompassed: thrombotic or inflammatory involvement of target vasculature; localized infections at puncture sites (e.g., tinea cruris); prior femoral/pelvic vascular surgery; pre-existing inguinal hernia; and peripheral artery disease.

D. Consent Procedure

All potential subjects that are identified by the chief anesthetist and/or designee that meet the inclusion/exclusion criteria will be given the opportunity to participate. Guardians/patients will be given the consent/assent during the screening visit. They will be given the opportunity to review the consent/assent and ask questions about the study. Guardians/patients will be asked to summarize in their own words what participation in this research study involves and that they are comfortable with the risks and benefits of participating in the research study. Any additional questions they have will also be answered by the investigator prior to signing the consent/assent. Once the consent/assent form is signed, a signed and dated copy of the authorization form will be provided to the subject and another copy placed in the participant's medical record.

E. Randomization and Blinding

Randomization was performed using a computer-generated random numbers table with a 1:1 allocation ratio. The allocation sequence was sequentially numbered and sealed in opaque envelopes by the corresponding author. The anesthesiologists opened the envelopes 10 minutes before the procedure commenced. Due to the nature of the intervention, full blinding of all study personnel was not feasible. However, to minimize bias, we attempted to blind the patients, research assistants, and data analysts. To achieve patient blinding, during the informed consent process, we described all interventions as femoral vein catheterization, and we explained that depending on group allocation, patients might receive different puncture trajectories.

F. Sample Size Calculation

PASS 2021 (NCSS, East Kaysville, UT, USA) was employed for sample size computation. A small, non-blinded, unpublished pilot study with 20 participants indicated that the puncture success rates for the lateral and orthogonal approaches were 90% and 60%, respectively. With a two-sided error of 0.05 and a power of 0.9, the sample size for each group was calculated as 42. To account for 20% attrition, we increased the sample size to 54 per group. In sensitivity analyses, even with conservative assumptions (risk ratio=1.3 or 30% attrition), the original sample size maintained over 80% power.

G. Statistical Analysis

Continuous variables were presented as mean (SD) or median (interquartile range, [IQR]), depending on whether the data was distributed normally or not. Categorical variables were presented as a number (percentage). The normal distribution of data was evaluated using the Shapiro–Wilk test, and the Levene method was used to test the homogeneity of variance. Differences in repeated measures variables were analyzed by repeated-measures analysis of variance. Continuous outcomes were analyzed with one-way analysis of variance (ANOVA) or Kruskal-Wallis test. Categorical variables were tested using $\chi 2$ test or Fisher's exact test. Time-to-event outcomes were illustrated using Kaplan-Meier curves. For a post hoc sensitivity analysis, we employed a logistic regression model. Statistical significance was defined as $P < 0.05$. Statistical analysis was conducted using SPSS v27.0 (SPSS Inc., Chicago, IL, USA).

H. Intervention and Study Procedures

During the access procedure, we adjust the patient to a supine position with a slight external rotation of the hips (the "frog-legged" position). Landmarks such as the anterior superior iliac spine, pubic symphysis, and inguinal ligament were identified. The femoral artery pulse was palpated 2-3 cm below the midpoint of the inguinal ligament and puncture was performed at 1-2 cm medial to the femoral artery. After sterile prep and a small subcutaneous dose of lidocaine, the needle was inserted steeply downward at a 30°-45°angle into the skin in different directions according to the patient's grouping and the timer was initiated. Crossover to the ultrasound-guided puncture occurred after three unsuccessful attempts. The timer was stopped when the puncture needle entered the target vessel or after three unsuccessful attempts. All patients underwent a puncture of the right femoral vein in this study.

Based on the relationship between the femoral vein and artery, Guan et al. classified the anatomy into four types (Fig. 3): Type I (vein parallel to artery without overlap),

Type II (vein medial to artery with lumen overlap $\leqslant 50\%$), Type III (vein posterior to artery with lumen overlap > 50%), and Type IV (vein lateral to artery) [9]. After the puncture catheterization, vascular ultrasonography was used to evaluate the femoral vasculature anatomy in the puncture area of each patient.

I. Measurements and Endpoints

The following parameters were systematically recorded for all enrolled patients:

(1) First-attempt Success Rate: Defined as the successful aspiration of venous blood through the needle on the initial skin puncture, without any needle withdrawal or redirection. This metric primarily reflects the precision of the initial puncture technique.
(2) Overall Success Rate: Defined as the ultimate successful aspiration of venous blood, achieved within a maximum of three puncture attempts or before the occurrence of any significant complication necessitating termination of the procedure.
(3) Number of Puncture Attempts: This refers to the total count of separate skin punctures required to achieve successful venous blood aspiration. Each withdrawal of the needle tip completely from the skin followed by a new puncture constitutes a new attempt. Minor redirections of the needle beneath the skin without complete withdrawal are not counted as separate attempts.
(4) Puncture Time: Measured in seconds, from the moment the needle first touches the skin until the successful aspiration of venous blood. The timer is stopped immediately upon confirmation of free venous blood flow into the syringe.
(5) Procedure-related Complications: All adverse events occurring during or immediately after the puncture procedure were meticulously documented. These include, but are not limited to accidental arterial puncture, hematoma formation, pneumothorax, etc.
(6) Anatomic Relationship between the Femoral Vein and Artery.

IV. DATE AND SAFETY MONITORING

Clinical research will formulate a corresponding data safety monitoring plan according to the size of the risk. In the implementation stage of clinical research, record all adverse events in detail, handle and track them properly until they are properly resolved or the condition is stable, and report serious adverse events and unexpected events to the ethics committee, competent authoritiesn and drug regulatory authorities in a timely manner as required; The principal investigator will periodically conduct a cumulative review of all adverse events, and if necessary, hold an investigator meeting to assess the risks and benefits of the study; We will arrange independent data monitors to monitor the research data, and high-risk studies will establish an independent data safety monitoring committee to monitor the accumulated safety data and efficacy data to make a decision on whether to continue the study.

V. FUNDING

This study was funded by New Quality Clinical Specialty Program of High-end Medical Disciplinary Construction in Shanghai Pudong New Area (2024-PWXZ-02); Pudong New Area Health System Leading Talent Training Program (PWR12024-07).

VI. INFORMATION CONFIDENTIALITY

Medical records will be kept in the hospital, the investigators and ethics committees will be allowed to access the patient's medical records. Any public reporting of the results of this study will not disclose the patient's personal identity.

VII. LITERATURE CITED

[1] C. Jenssen, B. Brkljacic, M. Hocke, A. Ignee, F. Piscaglia, M. Radzina, et al. EFSUMB Guidelines on Interventional Ultrasound (INVUS), Part VI - Ultrasound-Guided Vascular Interventions. Ultraschall Med 2016;37:473-6.

[2] S. Kabashneh, V. Singh and S. Alkassis. A Comprehensive Literature Review on the Management of Distal Deep Vein Thrombosis. Cureus 2020;12:e8048.

[3] R. J. Cho, D. R. Williams and J. W. Leatherman. Measurement of Femoral Vein Diameter by Ultrasound to Estimate Central Venous Pressure. Ann Am Thorac Soc 2016;13:81-5.

[4] M. Lamperti, D. G. Biasucci, N. Disma, M. Pittiruti, C. Breschan, D. Vailati, et al. European Society of Anaesthesiology guidelines on peri-operative use of ultrasound-guided for vascular access (PERSEUS vascular access). Eur J Anaesthesiol 2020;37:344-76.

[5] T. Hoffman, M. Du Plessis, M. P. Prekupec, J. Gielecki, A. Zurada, R. S. Tubbs, et al. Ultrasound-guided central venous catheterization: A review of the relevant anatomy, technique, complications, and anatomical variations. Clin Anat 2017;30:237-50.

[6] P. Kupó, R. Pap, L. Sághy, D. Tényi, A. Bálint, D. Debreceni, et al. Ultrasound guidance for femoral venous access in electrophysiology procedures-systematic review and meta-analysis. J Interv Card Electrophysiol 2020;59:407-14.

[7] J. F. Uhl, C. Gillot and M. Chahim. Anatomical variations of the femoral vein. J Vasc Surg 2010;52:714-9.

[8] B. Saugel, T. W. L. Scheeren and J. L. Teboul. Ultrasound-guided central venous catheter placement: a structured review and recommendations for clinical practice. Crit Care 2017;21:225.

[9] W. Guan, X. Li, K. Chen, Y. Yao and J. Liu. Anatomical variation of femoral vessels and ultrasound-guided femoral vein puncture for catheter ablation of arrhythmias. Pacing Clin Electrophysiol 2024;47:330-5.
