# Supplementary material for: A Comparison Between Two Different Directions of Landmark‐Guided Femoral Vein Puncture: A Prospective Randomized Controlled Trial
Source: Anesthesiol Res Pract. 2026 Apr 16;2026:9638063. doi: 10.1155/anrp/9638063 (PMC13267157; doi:10.1155/anrp/9638063)
Supplement: Supplementary file 3 — Supporting Information 3 Standard operating procedure (SOP): The standardized procedure protocol used to ensure consistent execution and data collection across all trial participants. [file ANRP-2026-9638063-s003.pdf]

Femoral Venous Cannulation SOP

(Version 1.1 | Effective Date: 2025-01-01)

1. Purpose

Standardize procedural steps to minimize operator-dependent variability in:

- Puncture time measurement
- Attempt counting
- Complication documentation

2. Scope

Applies to all femoral venous access procedures performed under this study protocol.

3. Procedure

3.1 Pre-Procedural Preparation

- Equipment Check:
- ➢ Ultrasound machine calibrated per manufacturer guidelines
- ➢ Triple-lumen central venous catheters (Arrow International, 7Fr)
- ➢ Video recording equipment initialized with synchronized timestamp
- Observer Assignment:
- ➢ Second independent observer presents during all procedures.
- ➢ Observer trained in Seldinger technique and study-specific documentation.

3.2 Procedural Execution

- Positioning and Local Anesthesia
- ➢ Supine position with slight hip abduction
- ➢ Skin Disinfection
- ➢ Locating the puncture site: FA pulse palpated 2-3 cm below inguinal ligament midpoint; puncture at 1-2 cm medial to FA.
- ➢ A small subcutaneous dose of lidocaine
- Skin Puncture:
- ➢ Operator verbalizes "Start" when needle first penetrates skin at inguinal ligament midpoint.
- ➢ Simultaneous video timestamp activation
- Puncture Directions:
- ➢ The needle was inserted steeply downward at a 30°-45° angle into the skin.
- ➢ The lateral approach: The puncture site located medial to the femoral artery

but the needle directed laterally at a medial-to-lateral angle of 15° – 30° .

- The orthogonal approach: The needle's direction was aligned with the medial aspect of the femoral artery and remained parallel to it.

- Attempt Termination Criteria:
  - Successful: Dark red, non-pulsatile blood return
  - Unsuccessful: ≥3 attempts; Accidental femoral artery puncture or other complications; Operator/observer joint decision based on safety concerns.

An "attempt" is defined as a discrete procedural event initiated by the first puncture of the skin in the inguinal region with a needle, continuing until either successful vascular access is achieved or mutually agreed termination by the operator and independent observer when further attempts are deemed to increase patient risk.

3.3 Post-Procedural Documentation

- Primary Data:
  - Operator records attempt counts in electronic case report form (eCRF).
  - Independent observer verifies via de-identified video review within 24h.
- Complications

4. Quality Control

- Monthly Audit:
  - 10% random cases reviewed by blinded external reviewers.
  - Discrepancies resolved by third-party arbitration.

5. References

- Ethics Committee Approval: 2024YS-280
- Equipment Manuals: Philips CX50 Ultrasound, Central Venous Catheterization Kit (Arrow International, 7Fr)
