# Supplementary material for: A Comparison Between Two Different Directions of Landmark‐Guided Femoral Vein Puncture: A Prospective Randomized Controlled Trial
Source: Anesthesiol Res Pract. 2026 Apr 16;2026:9638063. doi: 10.1155/anrp/9638063 (PMC13267157; doi:10.1155/anrp/9638063)
Supplement: Supplementary file 4 — Supporting Information 4 Preliminary exploratory study: The pilot study that preceded and guided the design of the main trial. [file ANRP-2026-9638063-s004.pdf]

# Preliminary Exploratory Study: Femoral Venous Cannulation Techniques

(January-March 2024)

## Study Design

1. Type: Single-center, non-blinded, alternating allocation pilot study
2. Sample Size: 20 emergency patients requiring femoral venous access
3. Groups:

1) Lateral Approach Group (n=10): The puncture site located medial to the femoral artery but the needle directed laterally at a medial-to-lateral angle of 15° –30° .
2) Orthogonal Approach Group (n=10): The needle's direction was aligned with the medial aspect of the femoral artery and remained parallel to it.

## Participant Characteristics

| Parameter | All Participants (n=20) |
|---|---|
| Mean Age (years) | 52.3 ± 15.7 |
| BMI (kg/m²) | 21.1 ± 3.8 |

**Inclusion Criteria:**

1) Requirement for emergent venous access
2) No local infection at puncture site

**Exclusion Criteria:**

1) Coagulopathy (INR >1.5)
2) Patients who declined to participate

## Outcome Measures

| Endpoint | Lateral Group (n=10) | Orthogonal Group (n=10) |
|---|---|---|
| **Primary** | | |
| Success rate | 9/10 (90%) | 6/10 (60%) |
| **Secondary** | | |
| Puncture time (s) | 48.5 | 52.0 |
| Hematoma incidence | 1/10 (10%) | 0/10 (0) |

## Limitations

1. Methodological constraints:

1) Non-randomized allocation may introduce selection bias
2) Single-center design limits generalizability

2. Data gaps: Lack of long-term complication follow-up (>30 days)
