# Supplementary material for: A Comparison Between Two Different Directions of Landmark‐Guided Femoral Vein Puncture: A Prospective Randomized Controlled Trial
Source: Anesthesiol Res Pract. 2026 Apr 16;2026:9638063. doi: 10.1155/anrp/9638063 (PMC13267157; doi:10.1155/anrp/9638063)
Supplement: Supplementary file 5 — Supporting Information 5 Subgroup analysis of procedural outcomes stratified by puncture approach and anesthesia type. [file ANRP-2026-9638063-s005.docx]

**Supplementary Table 1. Subgroup analysis of procedural outcomes stratified by puncture approach and anesthesia type**

| Approach | Anesthesia | n | First-attempt success, n(%[95%CI]) | Overall success, n(%[95%CI]) | Puncture time, median (IQR), s | Attempts (1/2/3) |
| --- | --- | --- | --- | --- | --- | --- |
| Orthogonal | General | 25 | 19 (76 [57 to 89]) | 20 (80 [61 to 91]) | 51 (45, 61) | 19/1/5 |
|  | Non-general | 29 | 9 (31 [17 to 49]) | 13 (45 [28 to 62]) | 77 (50, 81) | 9/2/18 |
| Lateral | General | 35 | 26 (74 [58 to 86]) | 32 (91 [78 to 97]) | 50 (46, 58) | 26/3/6 |
|  | Non-general | 19 | 15 (79 [57 to 91]) | 17 (89 [69 to 97]) | 49 (45, 60) | 15/1/3 |

Note: Data are presented as n (% [95% CI]) for categorical variables and as median (IQR) for puncture time. Formal statistical comparisons were not performed due to the exploratory nature of this subgroup analysis and the limited sample sizes in some subgroups.

Abbreviations: CI, confidence Interval; IQR, interquartile range.
